# Chin Augmentation and Treatment of Chin Retrusion with a Flexible Hyaluronic Acid Filler in Asian Subjects: A Randomized, Controlled, Evaluator-Blinded Study

**DOI:** 10.1007/s00266-023-03812-2

**Published:** 2024-02-05

**Authors:** Yun Xie, Hongyi Zhao, Wenyu Wu, Jinhua Xu, Bi Li, Sufan Wu, Kevin Chen, Torun Bromée, Qingfeng Li

**Affiliations:** 1 Department of Plastic and Reconstructive Surgery, Shanghai Ninth People’s Hospital, Shanghai Jiao Tong University School of Medicine, Shanghai, China; 2 Beijing Hospital, Beijing, China; 3 Huashan Hospital, Fudan University, Shanghai, China; 4 Peking University Third Hospital, Beijing, China; 5 Zhejiang Provincial People’s Hospital, Hangzhou, Zhejiang Province China; 6 Galderma Trading Co, Shanghai, China; 7 Galderma, Uppsala, Sweden

**Keywords:** Hyaluronic acid, Filler, Chin retrusion, Randomized controlled trial, Aesthetic improvement, Asian

## Abstract

**Background:**

Aesthetic improvement of the chin is increasingly requested by patients, including those of Chinese origin.

**Methods:**

A randomized, evaluator-blinded, no-treatment controlled study evaluated the effectiveness and safety of a flexible hyaluronic acid (HA) filler, Restylane^®^ Defyne^TM^ (HA_DEF_), in the correction of chin retrusion in a Chinese adult population over 12 months after treatment. On Day 1, subjects were randomized 3:1 into two groups, HA_DEF_ or delayed-treatment controls, and those in the HA_DEF_ group were administered treatment. An optional touch-up treatment was administered 1 month after treatment to obtain optimal chin augmentation. The initially untreated control group was offered delayed-treatment after 6 months (including 1-month touch-up).

**Results:**

HA_DEF_ was superior to no-treatment in improving chin retrusion according to the blinded evaluator at 6 months [Galderma Chin Retrusion Scale (GCRS) responder rate (≥ 1-point improvement from baseline) of 81% vs*.* 5% for untreated controls; *p *< 0.001, meeting the primary effectiveness objective. A majority of subjects maintained improvement at 12 months (61% in the HA_DEF_ group). All subjects reported satisfaction with results at 6 months after treatment with HA_DEF_ and aesthetic improvement rates per the global aesthetic improvement scale (GAIS) were high for 12 months following treatment, with an acceptable safety profile.

**Conclusions:**

These results demonstrated HA_DEF_ to be effective and safe for the correction of mild-to-moderate chin retrusion in Chinese subjects, confirming findings previously observed in a western population.

**Level of Evidence I:**

This journal requires that authors assign a level of evidence to each article. For a full description of these Evidence-Based Medicine ratings, please refer to the Table of Contents or the online Instructions to Authors www.springer.com/00266.

## Introduction

The chin profile is considered as an essential part of facial beauty and along with an expanding global filler market, there is also a rising demand for aesthetic improvement of this facial region [1]. Hyaluronic acid (HA) fillers allow for augmentation of facial tissues while avoiding surgical procedures and are frequently used in clinical practice to inject the chin in Chinese individuals. [2, 3] However, to our knowledge, no randomized and controlled clinical investigation has yet evaluated the effectiveness and safety of HA filler for this indication in China.

The study presented herein was the pivotal study to evaluate effectiveness and safety of Restylane^®^ Defyne^TM^ (HA_DEF_) in the correction of chin retrusion in a Chinese population. HA_DEF_ is approved in China for treatment of nasolabial folds since 2021, and recently (2023) also received the extended approval to include injection into the chin. The product is approved in the USA for use in the chin since 2021 [4], following demonstration of effective and safe use of HA_DEF_ for the chin indication in a US population [5]. HA_DEF_ is designed with OBT™/XpresHAn™ technology that enables distributed integration into the tissue and provides flexible support and contour enhancement. [6, 7]

## Methods

### Study Design

This randomized, evaluator-blinded, no-treatment controlled study (ClinicalTrials.Gov Number NCT03597256) enrolled five sites in China (from Beijing, Shanghai, and Zhejiang Province) and was conducted from October 2018 to September 2020. On Day 1, subjects were randomized 3:1 into two groups, HA_DEF_ or delayed-treatment controls, and those in the HA_DEF_ group were administered treatment. An optional touch-up treatment was administered at 1 month after treatment to obtain optimal chin augmentation. Effectiveness and safety were followed until 12 months after the last HA_DEF_ treatment. The initially untreated control group was offered delayed- treatment after 6 months (including a 1-month touch-up), and control subjects who received this treatment were then assessed for an additional 12 months for safety and effectiveness.

### Eligibility Criteria

Eligible subjects were adults 18 years or older of Chinese origin with mild-to-moderate chin retrusion. Key exclusion criteria included scars or deformities, disease, or lesions near or in the area to be treated; previous hypersensitivity to any injectable HA gel or to local anesthetics, history of severe allergies; previous tissue augmentation therapy with any permanent or semi-permanent filler; previous facial surgical therapy, laser treatment, or chemical peeling (below the level of the horizontal line from subnasale) within 6 months of study treatment; or use of neurotoxin or HA-based/collagen-based fillers (below the level of the horizontal line from subnasale) within 12 months of study treatment.

### Treatment

HA_DEF_ comprises 20 mg HA/mL and lidocaine hydrochloride 3 mg/mL and was injected using a 27G × ½” ultra-thin wall needle, primarily in the area defined as inferior to the lower lip, between the lines from oral commissure and pre-jowl sulcus. At each treatment session (initial treatment or optional touch-up), it was recommended to inject a maximum dosage of 2 mL in this area. One potential injection technique included administration of HA_DEF_ at one injection point at the most anterior portion of the chin with or without 1-point on each side of the chin at the discretion of Investigators. To obtain optimal results, an additional 2 mL could be injected in other areas of the chin at each treatment session. Injections could be given at more than one injection depth (mid to deep dermis, subcutis or supraperiostic zone), and with more than one injection method.

### Assessments

The primary effectiveness objective of this study was to evaluate whether aesthetic improvement of the chin (chin retrusion) at 6 months following injection with HA_DEF_ was superior to no-treatment. The corresponding primary effectiveness endpoint was percentage of responders [defined as at least 1-point improvement from baseline on the galderma chin retrusion scale (GCRS, a 4-point scale from no to severe retrusion) [8] assessed by a blinded evaluator] in the treatment group versus control group (6 months after last treatment or randomization, respectively).

Secondary effectiveness objectives were to evaluate chin retrusion, aesthetic improvement, and subject satisfaction. Specifically, secondary effectiveness endpoints comprised GCRS (% responders) to evaluate chin retrusion at other time points at 3, 9, and 12 months after last treatment by the blinded evaluator and at each follow-up visit for treating investigators (i.e., 1, 3, 6, 9, and 12 months after last treatment). Aesthetic improvement was based on a global aesthetic improvement scale (GAIS, a 5-point scale from worse to very much improved), assessed by the subject and treating investigator at each follow-up visit up to 12 months after treatment. The endpoint of GAIS was % responders, defined as having at least an “improved” score according to GAIS (i.e. including “improved”, “much improved,” and “very much improved”). Finally, a subject satisfaction questionnaire about treatment outcomes was administered at 3 and 6 months after last treatment.

Safety was evaluated based on adverse event collection throughout the study and pre-defined expected injection-related events recorded using subject diaries for two weeks after each treatment.

### Statistical Methods

Three analysis populations were defined for the study. They are the safety population (all subjects who were treated with HA_DEF_ or randomized to delayed-treatment control, and analyzed according to the as-treated principle), the full analysis set (FAS, all subjects who were treated with HA_DEF_ or randomized to delayed-treatment control, and analyzed according to the as-randomized principle), and the per protocol (PP) population (all FAS subjects who had no deviations that could affect evaluation of the primary variable). The primary endpoint (blinded evaluator GCRS at Month 6) was imputed using the baseline observation carried forward method. The FAS population was the primary population for all effectiveness analyzes, and the primary effectiveness analysis was repeated using the PP population.

This study was designed to confirm that the effectiveness in the treatment group is superior to the no-treatment control. The primary effectiveness variable was GCRS responder rates at month six, where the percentage of responders in the HA_DEF_ group was compared to the percentage of responders in the control group using Fisher's exact test at a significance level of 5%. The two-sided 95% CIs around the estimates of the percentage of responders for each group were calculated. Superiority was achieved if the 95% CIs for the difference between groups excluded 0, and the p-value was less than 0.05. The sample size was calculated to achieve 90% power to detect a difference between the groups. Also, Fisher’s exact test was used to compare response rates for GCRS at other time points. GAIS, satisfaction, and safety variables were summarized descriptively.

## Results

### Subject Disposition, Demographic, Baseline, and Injection Data

A total of 111 subjects were randomized to HA_DEF_ and 37 to the control group. Demographic data are presented in Table 1, and injection details are presented in Table 2. All subjects were treated on Day 1, and touch-up injections were administered at month 1 to 48 subjects in the HA_DEF_ group. A mean of 2.1 mL of HA_DEF_ was injected in the primary treatment area of the chin (*n*=111) and another 1.4 mL in other areas of the chin for augmentation (*n*=65) in the group randomized to HA_DEF_, including initial and touch-up treatments. In the control group, initial treatment occurred at 6 months, with similar volumes injected as in the treatment group.

**Table 1:** Demographic data and baseline characteristics

		HA_DEF_ (*n*= 111)	Control (*n*= 37)	Total (*n*= 148)
Age (years)	Mean (SD)	33.4 (8.0)	32.1 (7.1)	33.0 (7.8)
	Median	34.0	31.0	32.0
	Min, Max	21, 54	21, 52	21, 54
Gender *n* (%)	Female	105 (94.6%)	35 (94.6%)	140 (94.6%)
	Male	6 (5.4%)	2 (5.4%)	8 (5.4%)
Ethnicity *n* (%)	Han Chinese	109 (98.2%)	36 (97.3%)	145 (98.0%)
	Other^a^	2 (1.8%)	1 (2.7%)	3 (2.0%)
GCRS: blinded evaluator				
*n* (%)	1 – Mild	39 (35.1%)	9 (24.3%)	48 (32.4%)
	2 - Moderate	72 (64.9%)	28 (75.7%)	100 (67.6%)

^a^Mongolian, Man Chinese, the Hui nationality

**Table 2:** Volume (mL) of HA_DEF_ injected

		HA_DEF_			Control^a^		
		*N*	Mean (mL) ± SD	Range	*N*	Mean (mL) ± SD	Range
Primary treatment area of the chin	Initial	111	1.7 ± 0.4	(0.6, 2.0)	34	1.7 ± 0.4	(1.0, 2.0)
	touch-up	48	1.1 ± 0.5	(0.2, 2.0)	16	1.2 ± 0.6	(0.1, 2.0)
	total	111	2.1 ± 0.9	(0.6, 4.0)	34	2.2 ± 1.0	(1.0, 4.0)
Other areas of chin	Initial	61	1.1 ± 0.6	(0.1, 2.0)	22	1.1 ± 0.6	(0.2, 2.0)
	touch-up	30	0.8 ± 0.5	(0.1, 2.0)	14	1.0 ± 0.7	(0.1, 2.0)
	total	65	1.4 ± 1.0	(0.1, 3.9)	23	1.7 ± 1.3	(0.2, 4.0)

^a^In the control group, the initial treatment was at 6 months. 2 patients withdrew before 6 months, and 1 patient declined injection.

In the HA_DEF_ group, supraperiosteal-depth injections were administered in all but one subject (99%) at initial treatment and in all 48 subjects treated at touch-up; approximately 13% of treated subjects at each treatment occasion were also injected subcutaneously. In terms of the injection method, in the HA_DEF_ group, a bolus injection was administered at all initial and touch-up treatments in combination with fanning injection (in 21% and 8% of treated subjects, respectively) and linear retrograde threading (in 7% and 6% of treated subjects, respectively).

### Effectiveness

**Fig. 1.**
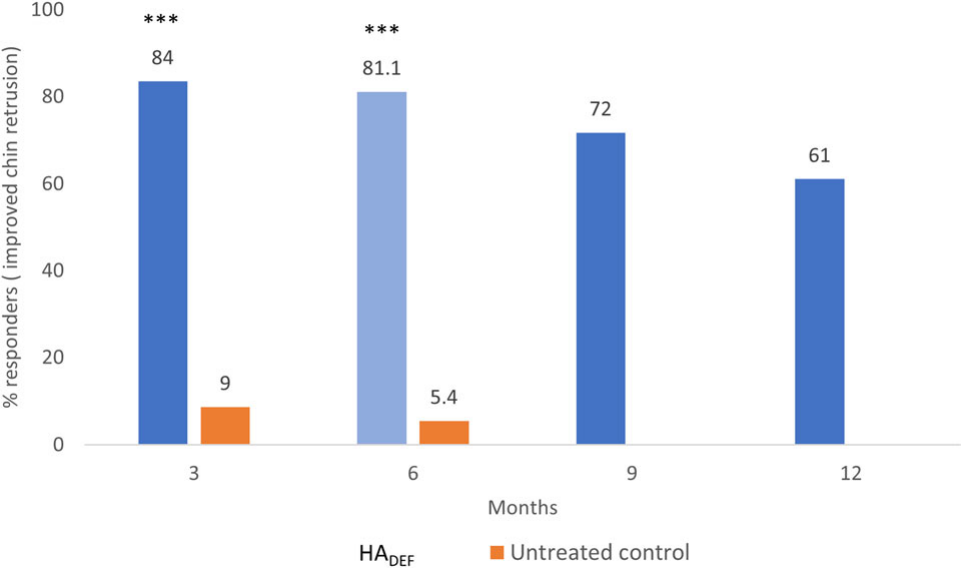
Chin retrusion responder rates (GCRS by blinded evaluator) (FAS). ***p<0.001 *vs.* control. FAS- full analysis set. Analysis based on FAS, and for the primary effectiveness endpoint (Month 6), missing values were imputed using the baseline observation carried forward (BOCF) method. Month 3, month 9, and month 12 are based on observed cases, i.e., no imputation of missing data was done for these time points.

Meeting the primary objective at 6 months, the HA_DEF_ group was superior to no-treatment in terms of improved chin retrusion according to the blinded evaluator, measured by the GCRS responder rate (81% vs*.* 5% for control; *p *< 0.001) (Fig. 1).

Moreover, secondary efficacy objectives supported the effectiveness of HA_DEF_ in terms of GCRS at other time points, GAIS, and subject satisfaction. At 3 months, a significantly greater proportion of subjects had improved GCRS scores in the HA_DEF_ group than in the control group as assessed by the blinded evaluator (84% vs*.* 9% for control; *p *< 0.001), and a majority of subjects maintained improvement at 12 months (61% in the HA_DEF_ group) (Fig. 1). Likewise, results were similar in GCRS assessments made by treating investigators (Table 3).

**Table 3:** Chin retrusion responder rates (GCRS, % of responders) (FAS, OC)

		Month 3	Month 6
Treating investigator, *n*/*N* (%)	HA_DEF_	92/109 (84.4%)	86/107 (80.4%)
	Control	0	0
	*p-*value	<0.001	<0.001

*n*number of responders; *N*number of subjects

*FAS* Full Analysis Set, *OC* observed cases

*P*-values for the difference in percentage of responders based on the Fisher’s exact test

**Fig. 2.**
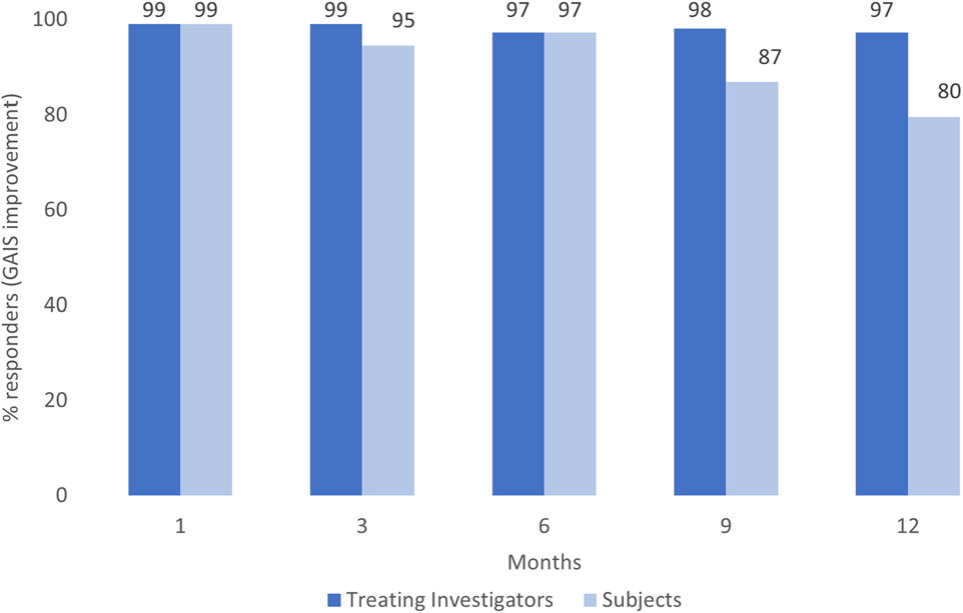
GAIS response rate over time in HA_DEF_ group, treating investigators and subjects (FAS, OC). FAS: full analysis set; OC: observed cases. A responder was a subject with a GAIS score of improved, much improved or very much improved.

**Fig. 3.**
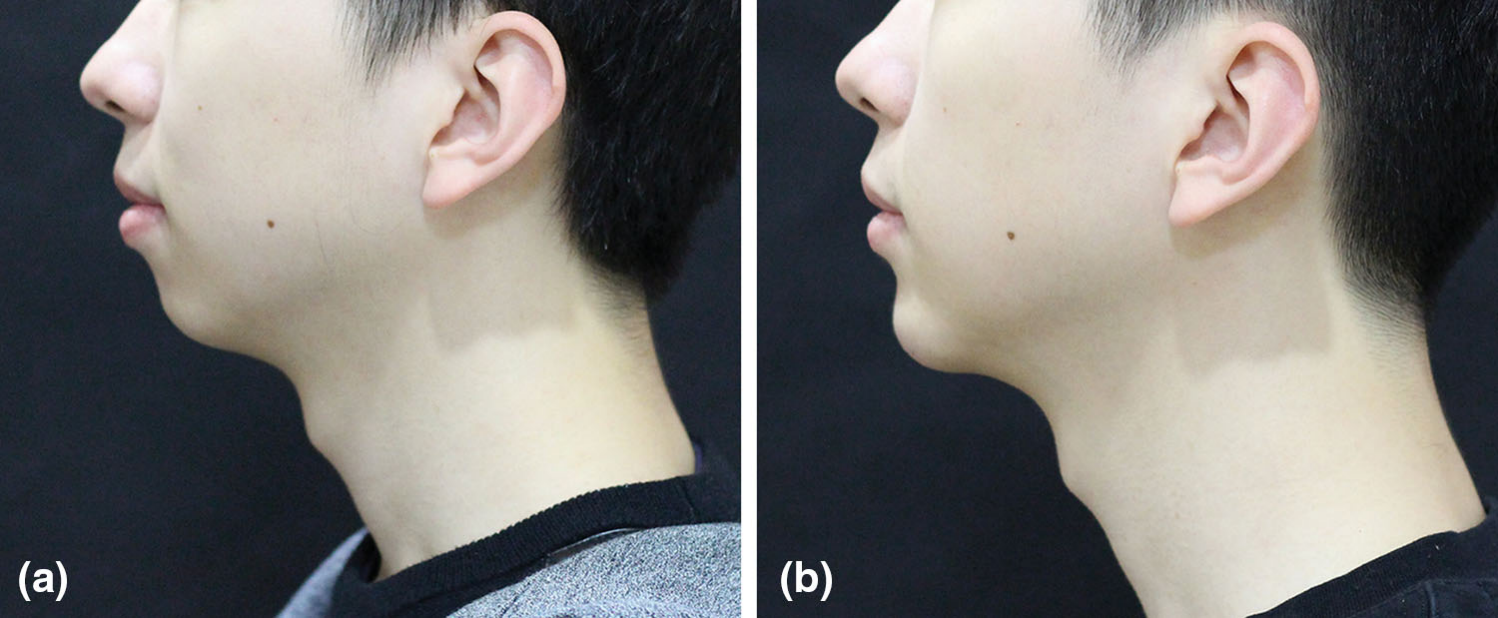
Subject photographs at (**a**) baseline (GCRS: 2—moderate) and, (**b)** 6 months (GCRS: 1—mild) after treatment with HA_DEF_. This 24-year-old male was randomized to the treatment group and received supraperiosteal bolus injections: 2 mL in the chin + 2 mL in other areas at initial treatment, and 1 mL in the chin + 1 mL in other areas at touch-up.

**Fig. 4.**
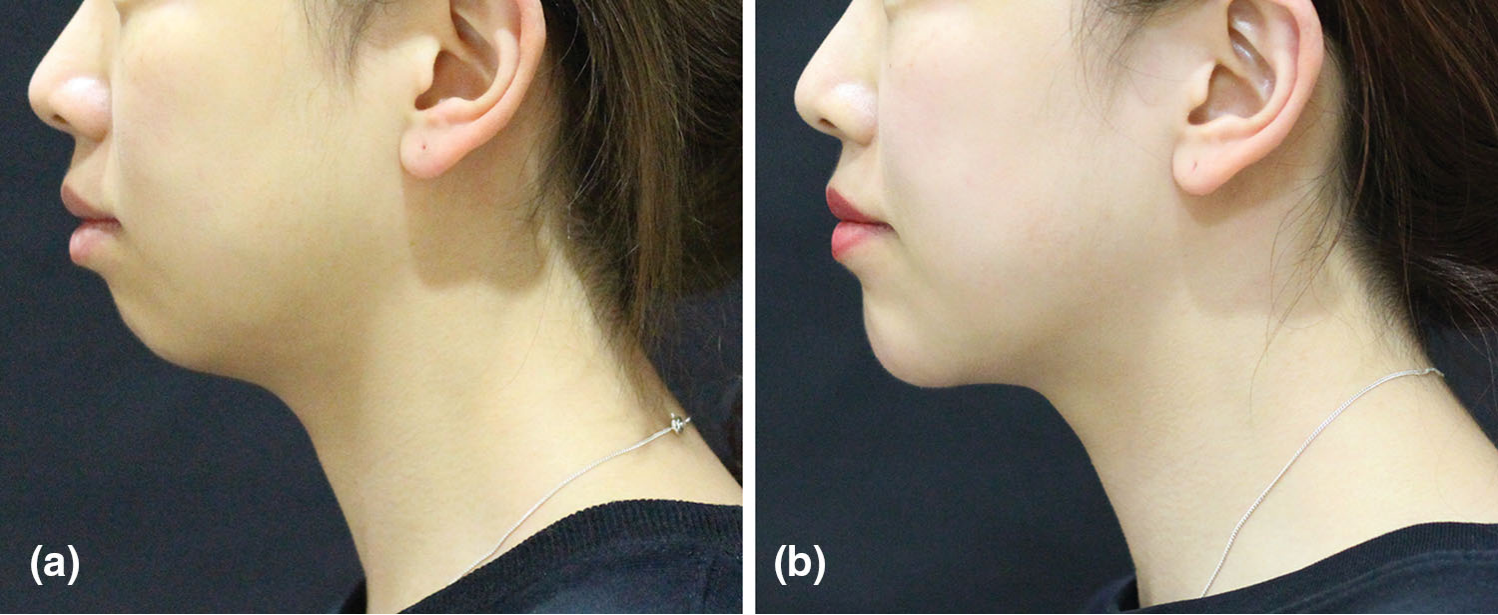
Subject photographs at (**a**) baseline (GCRS: 2—moderate) and **(b**) 6 months (GCRS: 1—mild) after treatment with HA_DEF._ This 23-year-old female was randomized to the treatment group and received supraperiosteal bolus injections: 2 mL in the chin + 2 mL in other areas at initial treatment, with no touch-up treatment.

Aesthetic improvement (GAIS) in the HA_DEF_ group remained high up to 12 months after treatment, as reported by both investigators (≥ 97% of subjects) and subjects (≥ 80%, Fig. 2). Subject photographs illustrating this improvement are shown in Fig. 3, 4.

**Fig. 5.**
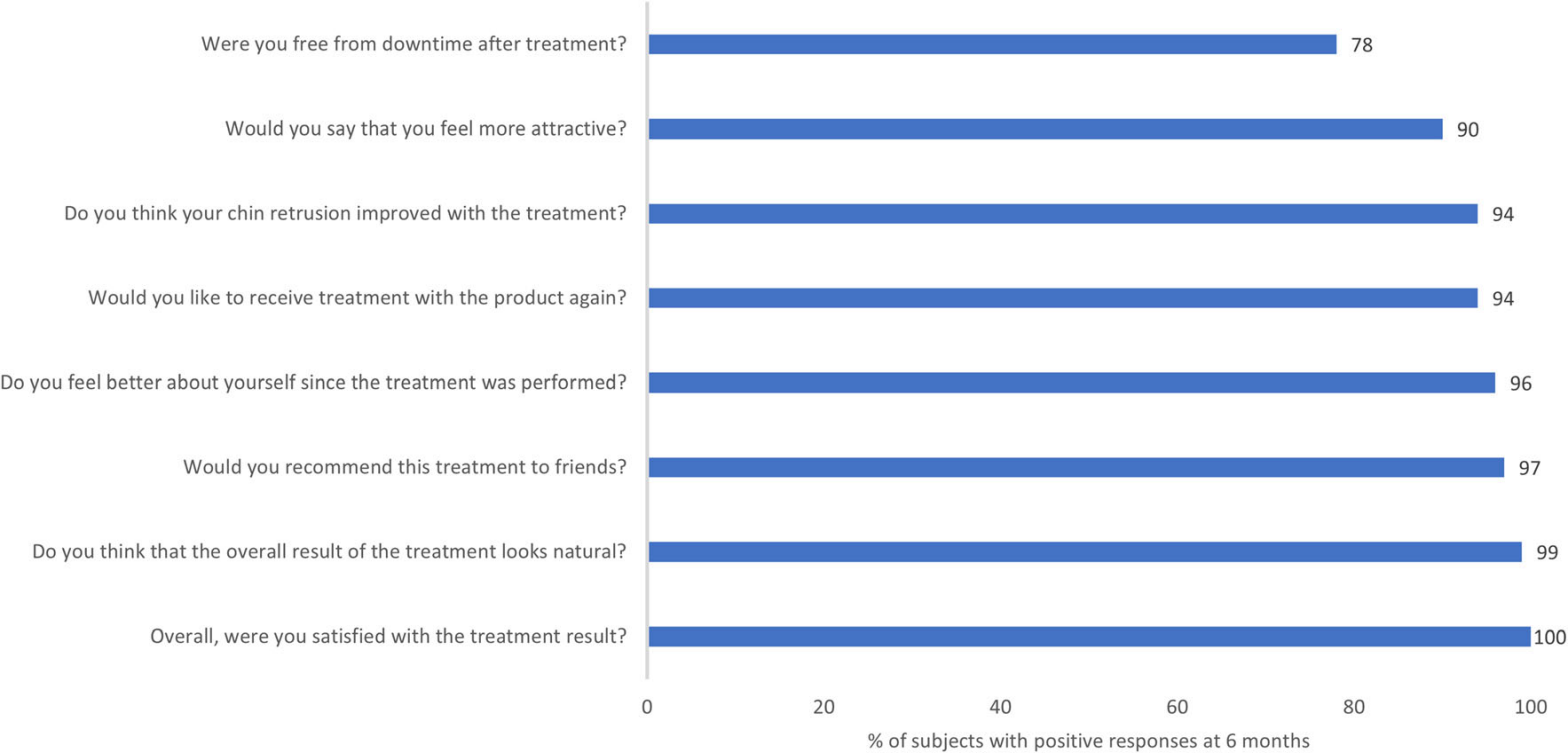
Subject satisfaction at 6 months in HA_DEF_ group

Subject satisfaction, assessed through 6 months, also remained high (Fig. 5). All subjects reported overall satisfaction with their results at 6 months after treatment with HA_DEF_. The majority reported natural-looking results (99%), felt more attractive (90%) and better about themselves (96%), thought their chin retrusion was improved (94%) with no downtime after treatment (78%), would like to receive HA_DEF_ again (94%), and would recommend it to others (97%).

Subjects in the control group who received treatment at 6 months showed similar responder rates at 12 months after treatment as in the HA_DEF_ group for both GCRS (blinded investigator) and subject-assessed GAIS (data not shown). For the GAIS evaluated by the treating investigator, the responder rate was slightly higher for the HA_DEF_ group (approximately 16 percentage units higher compared to the treated controls).

### Safety

Most subjects (97.2%) reported at least one pre-defined, expected post-treatment event after initial treatment in their 2-week diary, most commonly swelling (86%) and tenderness (82.5%), and most events were transient and mild or moderate in intensity. Treatment-related AEs were also mild or moderate, and consisted of injection site erythema (1.4%), injection site papule (0.7%), and syncope (0.7%). There were no serious treatment-related adverse events.

## Discussion

The primary objective of this study was met, HA_DEF_ led to superior aesthetic improvement of the chin compared to no-treatment in a chinese population at 6 months following injection. The improvement in chin retrusion as shown by GCRS scores was maintained in a majority of subjects (61%) for the 12 months of the study, with an acceptable safety profile. Furthermore, treatment with HA_DEF_ led to high subject satisfaction (assessed through 6 months), and high rates of aesthetic improvement for 12 months per GAIS scores. While further studies would be needed to assess the responder rates beyond 12 months, a duration of improvement of 12 months after the last treatment was shown for the majority of treated subjects in this study. A 6-12-month duration of effect is the generally expected longevity for HA filler treatments. Compared to permanent treatment options for correction of chin retrusion, e.g., silicone implants, augmentation of the chin via HA filler injection provides a non-surgical option for patients who do not want a permanent intervention or be exposed to the risks of surgery.

To our knowledge, this is the first randomized and controlled study conducted in an asian population to demonstrate effectiveness and safety of an HA filler for aesthetic treatment of the chin. This evidence is important to meet an increased demand for chin treatments [1] in China. For example, in a survey of over 1000 chinese individuals and over 700 chinese aesthetic practitioners, chin enhancement was the fourth most requested treatment (after square jaw/masseter reduction, wrinkle reduction, and nose enhancement) [9]. In addition, Liew et al. (2020) confirms that a well-projected chin is considered attractive among asian individuals. [10, 11]

The assessment scale used to measure chin retrusion in this study was the validated 4-point GCRS designed to measure a clinically relevant improvement. This scale has also been used in a previous US study (Marcus *et al*) [5] using the same HA product, HA_DEF_, and was therefore considered the most appropriate choice for confirming the effectiveness in this additional population.

In the prior mentioned US study (Marcus *et al*) [5], HA_DEF_ was evaluated using a randomized, evaluator-blinded, no-treatment control design for 48 weeks in adults with mild-to-moderate chin retrusion with similar outcomes as in the present study. The endpoints and definitions used were comparable to our study (i.e., GCRS responder rate was also ≥ 1-grade improvement). In terms of baseline severity, Marcus *et al* [5] presented that 63% of patients in the HA_DEF_ group in the US study had moderate retrusion, which was comparable to 65% in this study (Table 1).

The US study’s primary objective, to evaluate effectiveness of HA_DEF_ versus no-treatment at 3 months, was met with a significantly higher GCRS responder rate for HA_DEF_ (81%) than control (6%) (p<0.001), with maintained responder rates at 6 months. This mirrored the current study at both 3 months (84% vs*.* 9%) and 6 months (81% vs*.* 5%, respectively). In the US study, 5 comparable effectiveness in the primary endpoint was found across subgroups (e.g., different skin types, subject populations, genders and age groups; about 6% of patients included were asian). This is consistent with effectiveness seen in asian patients in this study, supporting that HA_DEF_ appears to be suitable for different populations. GCRS responder rates remained slightly higher in the HA_DEF_ group at 12 months (74% vs*.* 11% for control; *p *< 0.001) in the US study, and this reached 61% in our study. Nevertheless, high aesthetic improvement and subject satisfaction were found in both studies.

Overall, results of this study demonstrated HA_DEF_ to be effective and safe for the correction of mild-to-moderate chin retrusion in chinese subjects over 18 years of age. This study confirms the findings previously observed in a western population and supports expanded use of this HA filler in the chin in an asian population.
